# Intermittent Pneumatic Compression for the Treatment of Lower Limb Lymphedema: A Pilot Trial of Sequencing to Mimic Manual Lymphatic Drainage Versus Traditional Graduated Sequential Compression

**DOI:** 10.1089/lrb.2021.0025

**Published:** 2022-10-20

**Authors:** Nyree Dunn, Edgar M. Williams, Gina Dolan, Jane H. Davies

**Affiliations:** ^1^ Faculty of Life Sciences, University of South Wales, Pontypridd, United Kingdom.; ^2^ Huntleigh Healthcare, Cardiff, United Kingdom.

**Keywords:** lymphedema, intermittent pneumatic compression, leg volume, quality of life, decongestive lymphatic therapy

## Abstract

**Background:**

Recent advances in technology have allowed intermittent pneumatic compression (IPC) devices to develop so that their function mimics the process and principles of manual lymphatic drainage (MLD); however, research into the effectiveness of such devices is lacking. This study aimed to investigate the effectiveness of a patented IPC technique designed to mimic MLD (the LymphAssist), compared with a typical sequential IPC regimen.

**Methods and Results:**

Forty patients with a confirmed diagnosis of lower limb ISL (International Society of Lymphology) stage II or III lymphedema were recruited into this three-phased study. A bilateral leg volume assessment and quality-of-life assessment were completed at four clinic visits across the course of the study. The LymphAssist IPC regimen was significantly more effective in reducing distal leg volume than the sequential mode (mean volume reduction: 230 ± 135 mL vs. 140 ± 84 mL, respectively, *p* = 0.01). Improvements in leg volume were transient as both groups demonstrated a rebound or increase in volume during the washout period (LymphAssist: 238 ± 168 mL, sequential: 276 ± 158 mL, *p* = 0.3). Overall, IPC was effective in improving quality-of-life scores (mean reduction: 10 ± 11, *p* < 0.001).

**Conclusion:**

IPC is effective in reducing limb volume and improving quality of life for patients with lower limb lymphedema. IPC that mimics the MLD process has been shown to be more effective in reducing leg volume compared with traditional sequential IPC in the distal aspect of the leg. The increase in leg volume observed after discontinuation of IPC suggests that regular treatment is required to maintain its associated effects.

Clinical Trial Registration Number: NTC 03856281.

## Introduction

Decongestive lymphatic therapy (DLT) is well established as the gold standard treatment for lymphedema. While the individual components (compression, manual lymphatic drainage [MLD], exercise, and skin care) of DLT remain at the forefront of patients' treatment plans, adaptations to these plans are becoming more popular and include adjunctive interventions such as intermittent pneumatic compression (IPC) that can be incorporated into patient self-management regimens.^1^

IPC is a simple therapy where garments consisting of pneumatic cuffs are connected to a pump and applied to the limb. Typically, the simplistic technology of IPC is effective as it mimics the intermittent compression of lower limb vasculature that contracts while walking, thus ascending fluid up the limb during its application in lymphedema. IPC technology has evolved from single chamber garments in the 1950s to multichamber garments and advanced pumps that provide sequential or peristaltic compression in an ascending pattern up the limb, varying in timing cycles and pressure amounts, ranging from low-pressure slow-inflation to high-pressure rapid-inflation devices.^2,3^

The value of IPC has been reinforced with the majority of publications evaluating the efficacy of IPC in breast cancer-related lymphedema.^4–8^ Improvements were seen in limb volume and physical and emotional status (*n* = 324), using water displacement and circumferential measurements to record objective changes. Treatment duration ranged from 10 days to 12 weeks, with a significant reduction in limb volume found in each study (ranging from −5.8% to −45.3%).

An evidence base for lower limb lymphedema is limited by a scarcity of randomized trials and published data. However, the short-term effects of IPC have been investigated by Modaghegh and Soltani^9^ and were proven to be beneficial after 43 hospitalized patients received sequential IPC for 8 hours a day over 48 hours (total treatment time 16 hours). Treatment pressures were prescribed at 80–120 mmHg, which resulted in a mean edema reduction of 75%.

Furthermore, Taradaj et al.^3^ recruited 81 patients who were randomized into three treatment groups and treated using multilayered bandaging and MLD. Treatment was prescribed once a day, three times a week, for a total of 4 weeks. Patients in group A (treatment pressure: 120 mmHg) and group B (treatment pressure: 60 mmHg) also received sequential IPC before bandaging and MLD for 45 minutes, and those in group C received bandaging and MLD alone. Patients in group A displayed the biggest limb volume reduction of 38.45%, group B had a reduction of 13.12%, and finally, group C had a reduction of 11.89% (*p* < 0.01).

Technological advances in recent years have led to the development of IPC devices that incorporate complex compression regimens that have been designed to mimic the process and principles of MLD. Such regimens incorporate multichamber garments, which inflate and deflate in various patterns and pressures and apply compression in a proximal to distal cycle.^10^ However, there are few studies to support the efficacy of such regimens.^7,11^

The primary aim of this study was to compare the effectiveness of the LymphAssist IPC regimen, a technique designed to mimic the MLD process, against a sequential IPC regimen using the Hydroven 12 (Huntleigh Healthcare, United Kingdom) in reducing leg volume.

## Methods

### Design and ethics

**FIG. 1.**
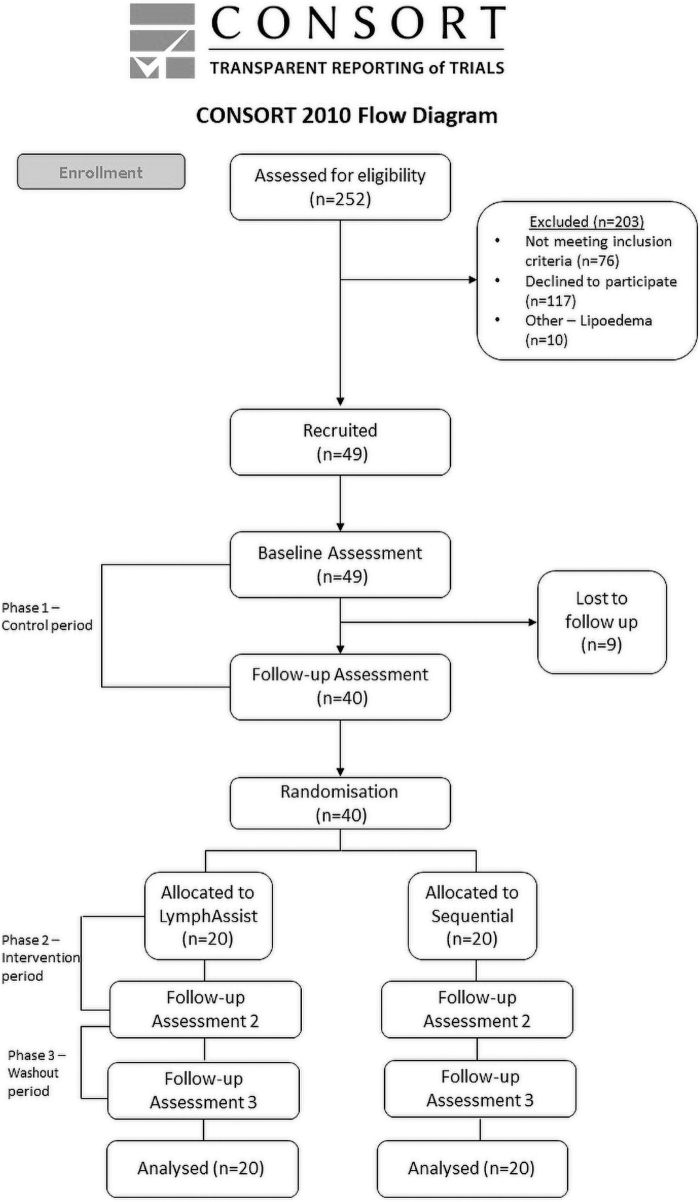
Consort flow diagram/study design.

A participant-blinded, randomized controlled trial (RCT), which incorporated three 5-week phases (Fig. 1), was designed to build on an earlier feasibility study, which utilized IPC delivered by the LymphAssist compared with a control group.^12^ Ethical approval for this study was obtained from the appropriate local National Health Service Research Ethics Committee (LREC No. 18/WA/0114) and registered with ClinicalTrials.gov.

### Participants

Participants (*n* = 40) with primary (*n* = 1) or secondary (*n* = 39) lymphedema were recruited from two outpatient lymphedema clinics in the South East Wales area.^12^ All participants had a confirmed diagnosis of stage II or III lower limb lymphedema as defined by the International Society of Lymphology; 33 participants had bilateral lower limb disease and 7 had unilateral.^13^ All participants provided written informed consent. The pragmatic design of the study meant that standard care for each participant could have varied in terms of compression garments, exercise, and skin care.

### Compression regimen

The Hydroven 12 professional system comprises a pump and a multichamber garment capable of delivering three types of treatment options ranging from a pressure of 15 to 120 mmHg for 35 minutes. For this study, pressures of 40 mmHg were used, and two regimens were selected and locked into the device: either the LymphAssist cycle—designed to mimic MLD or sequential IPC.

### Intervention

Participants were randomized using block randomization and were blinded to which regimen they would receive.^14^ Participants then received a 12-chamber leg garment plus either a Hydroven device locked into the LymphAssist cycle or a Hydroven device locked in to apply graduated sequential compression. The participants were instructed to use the device twice daily at a pressure of 40 mmHg at home, alongside their standard lymphedema treatment for 5 weeks.

### Outcome measures

The primary outcome evaluated during each phase at each clinic visit was leg volume. A bilateral limb assessment was undertaken, which included leg volume measurement using a circumferential tape measure method; a nonstretch tape measure (Medi, Germany) was used to measure leg circumference at 40 mm intervals from the top of the malleolus to a significant clinical end point that was taken from the patient's medical notes. The leg circumference measures were used to calculate the volume of the 40 mm leg segments using simple software (LymCalc V 4.0, United Kingdom) where leg volumes are calculated by adding together segments.^12,15^ Volume data derived from circumferential measurements were analyzed according to the aspect of the leg, namely:

- The distal aspect—from ankle to knee
- The proximal aspect—from knee to thigh
- Full leg—distal+proximal

Secondary outcomes also included a quality-of-life assessment using a condition-specific tool, the Lymphedema Functioning, Disability, and Health Questionnaire for Lower Limb Lymphedema (The Lymph-ICF-LL)^16^ by comparing scores across the study period. The Lymph-ICF-LL is a validated questionnaire, which uses analogue scales from 0 to 10 to evaluative 28 questions about impairments in function, activity limitations, and participation restrictions in patients with lower limb lymphedema.

The questionnaire has five domains: physical function, mental function, general tasks/household activities, mobility activities, and life domains/social life. The total score was calculated as follows: (sum of scores on questions/total number of answered questions) × 10. In the same way, a score was determined for each of the five domains. The total score and the domain scores ranged from 0 to 100, a higher score denotes a poorer quality of life.^16^

### Statistical analysis

Comparison of means and correlation between the groups were performed using computer software (IBM SPSS, version 22; New York, and Sigmaplot, Version 14; Sysstat, United Kingdom). Analysis of variance was used to test for significance between and within groups. Statistical significance was set at *p* < 0.05. Categorical data were analyzed using Pearson's chi-squared test.

## Results

### Baseline characteristics and those lost to follow-up

From July 10, 2018, to July 9, 2019, 49 participants were recruited into the study; 9 participants were lost to follow-up before the intervention period. There were no significant differences between the study population and those who were lost to follow-up, expect for gender ratio (Table 1). Demographics of the completed study population had a mean age of 59 ± 11 years (range 44–77 years), mean weight 100 ± 29 kg, mean body mass index of 35 ± 8, and the percentage ratio of female to male participants was 80:20 (*n* = 32:8). There were no significant differences between the intervention groups at baseline (Table 2).

**Table 1.** Population Demographics at Baseline of Those Who Completed the Study Compared with Those Who Were Lost to Follow-Up

	Completed (*n* = 40)	Lost to follow-up (*n* = 10)	*p*
Age, years	59 ± 11	61 ± 13	0.7
Gender, F:M, %	80:20 (32:8)	44:56 (4:5)	0.04
Weight, kg	100 ± 29	97 ± 26	0.9
Height, cm	167 ± 10	168 ± 9	0.4
BMI	35 ± 8	33 ± 10	0.2
Stage II:III, %	93:7 (37:3)	100:0 (9:0)	0.6

BMI, body mass index; F, female; M, male.

**Table 2.** Population Demographics at Baseline According to the Intervention Group

	LymphAssist (*n* = 20)	Sequential IPC (*n* = 20)	*p*
Age, years	61 ± 10	60 ± 10	0.6
Gender, F:M, %	74:26 (15:5)	85:15 (17:3)	0.4
Weight, kg	104 ± 31	97 ± 27	0.3
Height, cm	167 ± 12	166 ± 9	0.3
BMI	36 ± 9	34 ± 7	0.4
Stage II:III, %	80:20 (19:1)	85:15 (17:3)	0.08
Affected lymphedema volume, mL	8972 ± 2997	8692 ± 2710	0.6

IPC, intermittent pneumatic compression.

### The LymphAssist cycle

**FIG. 2.**
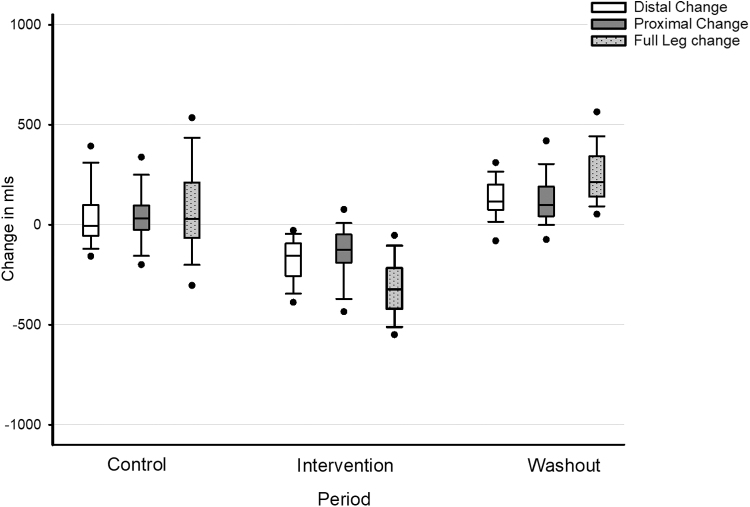
Changes in leg volume (in milliliters) across each study period in the LymphAssist group. Notation: The *black bar* represents the means, the *box* represents the 95% confidence intervals, the *error bars* represent the standard deviation, and the *circles* are outliers.

Participants in the LymphAssist group exhibited no significant volume changes in any aspect of the leg following standard lymphedema treatment only (the control period) (Fig. 2). However, significant decreases in volume were observed following the addition of IPC (during the intervention period): in the distal aspect, a loss of 230 ± 135 mL (*p* < 0.001), 124 ± 118 mL in the proximal leg (*p* < 0.001), and a total loss of 357 ± 167 mL overall (*p* < 0.001). In phase 3, at the end of the washout period, there was a significant rebound in fluid increasing by 130 ± 117 mL in the distal limb (*p* < 0.001), 100 ± 105 mL in the proximal limb (*p* < 0.001), and 238 ± 168 mL overall (*p* < 0.001).

### Sequential IPC

**FIG. 3.**
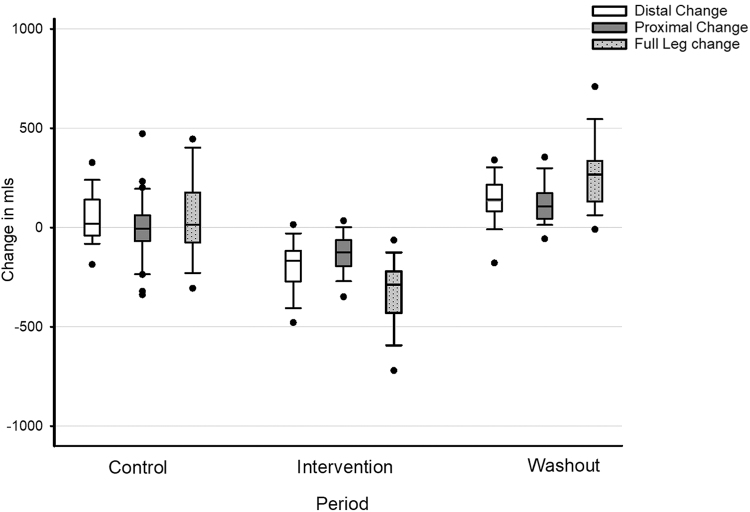
Changes in leg volume (in milliliters) across each study period in the sequential group. Notation: The *black bar* represents the means, the *box* represents the 95% confidence intervals, the *error bars* represent the standard deviation, and the *circles* are outliers.

As with the LymphAssist group, participants in the sequential IPC group also exhibited no significant changes in any aspect of the leg volume during the control period (Fig. 3). Significant decreases in volume were observed following the addition of IPC: in the distal limb of 140 ± 84 mL (*p* < 0.001), 150 ± 158 mL in the proximal limb (*p* < 0.001), and 287 ± 140 mL overall (*p* < 0.001). At the end of the washout period, there was a significant rebound in fluid increasing by 137 ± 111 mL in the distal limb (*p* < 0.001), 138 ± 128 mL in the proximal limb (*p* < 0.001), and 276 ± 158 mL overall (*p* < 0.001).

### A comparison of IPC modes

A comparison of absolute changes in volume showed that the LymphAssist was significantly better at reducing leg volume than the sequential cycle regimen in the distal part of the leg, a reduction of 230 ± 135 mL versus 140 ± 84 mL, respectively (*p* = 0.01). There were no differences in the proximal aspect of the leg, those in the sequential group had a reduction in volume of 150 ± 158 mL versus 124 ± 118 mL in the LymphAssist group (*p* = 0.7) (Table 3).

**Table 3.** Effect of Intermittent Pneumatic Compression on Leg Volume Reduction: A Comparison of the Change in Milliliters from Both Modes

	LymphAssist (*n* = 37 legs)	Sequential (*n* = 36 legs)	*p*
Distal	230 ± 135	140 ± 84	<0.01
Proximal	124 ± 118	150 ± 158	0.7
Full	357 ± 167	287 ± 140	0.06

Data are presented as mean ± standard deviation.

### Quality of life

The Lymph-ICF-LL total scores did not change after the control phase with a mean score of 40 ± 22 (*p* = 0.6). Following the IPC use, quality of life significantly improved, with an average score of 32 ± 22 (*p* < 0.001). Finally, once IPC was removed, scores rebounded to 43 ± 22 (*p* = 0.01) indicating that quality of life was significantly lower once IPC was removed. There were no statistical differences in scores between the interventions falling by 8 ± 11 and 6 ± 9 with the LymphAssist and sequential IPC regimens, respectively (*p* = 0.5). The biggest improvements were observed in the physical domain, with scores significantly reducing by 16 (±16) from an average score of 38 ± 21 to 26 ± 20 (*p* < 0.001).

## Discussion

The study supports the value of IPC as an adjunctive treatment for managing lower limb lymphedema, with significant reductions in volume following the addition of IPC and significant increases or “rebounds” in volume following its removal. During the control period, there were no significant changes in leg volume. All participants were in compression hosiery during the study period, hence in stage II of complete decongestive therapy, the maintenance phase. Therefore, only small changes in leg volumes were expected as good compliance with compression garments had already been established.

Following the use of IPC, the LymphAssist was significantly better at reducing distal leg volume than the sequential cycle mode, with a mean reduction of 230 ± 135 mL compared with 140 ± 84 mL, respectively (*p* < 0.001) Full volumes showed that the LymphAssist mode resulted in larger reductions in volume 357 ± 167 mL compared with the sequential mode, 287 ± 140 mL; however, this was not significant (*p* = 0.06).

Few studies have compared the efficacy of an advanced pneumatic compression device, which mimics the MLD process to a sequential IPC device; however, Fife et al. compared two such devices with a cohort of participants with arm lymphedema. IPC was applied an hour a day for 12 weeks at a pressure of 30 mmHg. Eighteen participants were assigned to each group with those using an advanced device experiencing significantly better reductions in volume of 118 ± 170 mL compared with an increase of 6 ± 216 mL with those using a simple device (*p* = 0.01). The simple compression devices consisted of a pump and a three-chamber garment, whereas the advanced device included a multichamber garment consisting of three parts, including the arm, adjacent chest, and truncal quadrant.^7^

However, there are differences in treatment regimens in terms of discrepancies in garments, mean that a direct comparison of treatment cycles is difficult. Nevertheless, the effectiveness of MLD mimicking devices is supported by a study conducted by Muluk et al., one of the largest studies to assess the efficacy of such devices in lower limb lymphedema patients. IPC was utilized for 60 ± 27 days with the study findings being comparable to those of the current study, with 90% of the 196 participants recruited into the study achieving a mean reduction in volume of 8% (*p* < 0.001). In this group, 35% had a reduction greater than 10%.^11^

Further support of IPC for treating lower limb lymphedema is provided by Zaleska et al., who recruited 18 unilateral participants receiving sequential IPC at pressures of 100–120 mmHg. Over a 3-year period, participants enjoyed reductions in circumferential measurements at both the calf and the thigh. After the first month, calf measurements reduced by 2.3% ± 3.9% and 2.1% ± 3.8% in the thigh (*p* < 0.05); similar reductions were observed in the current study over a similar period, with a mean volume reduction of 323 ± 158 mL in all participants, which translates to 4% ± 2% reduction; however, this was achieved with much lower pressures of 45 mmHg. Reductions in circumferential measurements were maintained after 12 months in the study by Zaleska et al., although the benefit started to plateau following the first year of continual IPC use.^17^

The current study did not assess the long-term benefits of IPC; however, the results of the washout period in the current study, which found significant rebounds in volume, support the findings of Zaleska et al. and suggest that the regular use of IPC is required. Those in the LymphAssist group significantly rebounded by an increase in 238 ± 168 mL (*p* < 0.001) and the sequential group by 276 ± 158 ML (*p* < 0.001). Those in the sequential group, on average, rebounded slightly higher than those in the LymphAssist group; however, this was not significant (*p* = 0.3).

However, some evidence suggests that it is unclear whether IPC has any additional benefits when used in combination with DLT.^18^ A review of the clinical evidence conducted by Tran and Argaez found the effectiveness of IPC for volume reduction; a systematic review and two RCTs found no significant differences between DLT combined with IPC compared with DLT alone. However, one RCT and the systematic review investigated the effects of IPC in breast cancer-related lymphedema patients.

The second RCT was a study by Taradaj et al., which found over 4 weeks, in 81 participants, those who used sequential compression at a pressure of 120 mmHg had a significantly greater volume reduction of 38% compared with 13% in those who applied IPC at a pressure of 60 mmHg and 12% in the control group who received MLD and bandaging only (*p* < 0.01). Results showed no significant difference between the control group and those who used IPC at 60 mmHg, suggesting that higher pressures are more effective for reducing leg volume.^3^ Furthermore, participants in the study by Taradaj et al. utilized IPC alongside bandaging, whereas the current study aimed to investigate the effectiveness of IPC in the home, in phase II of treatment.

This study is one of the few studies to consider the effects of IPC on separate sections of the limb, the distal and proximal sections. Distally, the LymphAssist mode was significantly better at reducing leg volume (230 ± 135 mL) than the sequential cycle mode (140 ± 84 mL) (*p* ≤ 0.01). However, proximally, there were no differences between the two treatment modes with those in the LymphAssist group recording an average volume loss of 124 ± 118 mL and 150 ± 158 mL in the sequential group (*p* = 0.7).

Collins et al. used computed tomography to monitor the response of compression therapy utilized for 12 weeks in 27 unilateral participants. Following 12 weeks of treatment, both the proximal and distal aspects of the affected limb had a significant decrease in overall standardized cross-sectional area over the study period, with an average decrease of 26% distally (*p* ≤ 0.01) and 9% proximally (*p* = 0.02) in the subcutaneous alone. The difference between the whole limb and the muscle mass was used to provide an absolute measurement of the subcutaneous compartment and therefore provide a more accurate measure of reduction in fluid.^19^

The psychosocial consequences of lymphedema are often underrated; however, the emotional impact can be just as detrimental as the physical symptoms.^20^ A systematic review conducted by Fu et al. found that 74% of people said that their lymphedema had a negative effect on their daily living, with self-care being so time-consuming identified as a key factor.^21^ In the current study, following the intervention period, significant improvements in quality of life were seen with an average score of 32 ± 22 compared with 40 ± 22 at the end of the control period (*p* < 0.001).

Once IPC was removed, quality-of-life scores returned to those observed at baseline 43 ± 22 (*p* = 0.01). Both IPC modes were successful at improving patient-reported quality-of-life scores especially the symptomatic feelings that are associated with lymphedema such as pain and heaviness. These results suggest that IPC is an effective treatment in improving patient-reported quality-of-life, which could in turn have a positive impact on the physical ailments, social relationships, and self-confidence issues experienced by those with lymphedema reported by Fu et al.

However, patient acceptance of IPC is essential for its effectiveness in improving lymphedema symptoms. Blumberg et al. also found improvements in quality of life associated with IPC use, which were observed by 100 participants utilizing IPC for an average of 12.7 months, 5.3 times per week. Post-IPC questionnaires were administered, and results found that 100% of participants reported symptomatic improvements. Fifty-four percent of participants felt that their symptoms had greatly improved and 90% would recommend the use of IPC to others. Following the observed reduction in limb volume and improvements in quality of life in the current study, one could assume that IPC was greatly accepted by the participants.^22^

### Limitations

The main limitation of this study concerns the small sample size of the intervention groups, which only represents a small sample of the South East Wales population; it is, however, of a similar size to other existing IPC studies. Another limitation relates to the pragmatic study design meaning that differences in standard care were not controlled for during the study. However, pragmatic studies are designed to mimic usual clinic practice rather than in a controlled well-designed setting, which would allow the investigation of applicability and generalizability of IPC, thus an investigation into whether IPC works in real life.^23^

The majority of study participants had secondary lymphedema; hence, the effectiveness of MLD-based IPC for patients with primary lymphedema should provide a focus for future research. Finally, while this study did include a 5-week washout period, the long-term effects of IPC were not investigated.

## Conclusion

This study adds to the evidence base regarding the efficacy of IPC for the treatment of lower limb lymphedema. Results suggest that the LymphAssist is more effective in reducing leg volume compared with sequential IPC. Furthermore, this study confirms the practicality of IPC as a home-use treatment, which fosters patient empowerment as patients gain more control over their lymphedema management.

## References

[1] Wigg J, Lee N. Redefining essential care in lymphoedema. Br J Community Nurs 2014; 20:24–27. 10.12968/bjcn.2014.19.sup4.s20 24704751

[2] Feldmen JL, Stout NL, Wanchai A, Stewart BR, Cormier JN, Armer JN. Intermittent pneumatic compression therapy: A systematic review. Lymphology 2012; 45:13–25. 22768469

[3] Taradaj J, Rosinczuk J, Dymarek R, Halski T, Schneider W. Comparison of efficacy of the intermittent pneumatic compression with a high-and-low pressure application in reducing the lower limbs phlebolymphedema. Ther Clin Risk Manag 2015; 11:1545–1554. 26504396 10.2147/TCRM.S92121 PMC4603726

[4] Szuba A, Achalu R, Rockson SG. Decongestive lymphatic therapy for patients with breast carcinoma-associated lymphedema. A randomized, prospective study of a role for adjunctive intermittent pneumatic compression. Cancer 2002; 95:2260–2267. 12436430 10.1002/cncr.10976

[5] Wilburn O, Wilburn P, Rockson SG. A pilot prospective evaluation of a novel alternative for maintenance therapy of breast cancer associated lymphedema. BMC Cancer 2006; 6:84–94. 16571129 10.1186/1471-2407-6-84 PMC1440867

[6] Ridner SH, McMahon E, Dietrich MS, Hoy S. Home-based lymphedema treatment in patients with cancer-related lymphedema or noncancer-related lymphedema. Oncol Nurs Forum 2008; 35:671–680. 18591171 10.1188/08.ONF.671-680

[7] Fife CE, Davey S, Maus EA, Guilliod R, Mayrovitz HN. A randomized controlled trial comparing two types of pneumatic compression for breast cancer-related lymphedema treatment in the home. Support Cancer Care 2012; 20:3279–3286. 10.1007/s00520-012-1455-2 PMC3480585 22549506

[8] Moattari M, Jaafari B, Talei A, Tabatabaee H, Piruzi S, Tahmasebi S. The effect of combined decongestive therapy and pneumatic compression pump on lymphedema indicators in patients with lymphedema secondary to breast cancer treatment: A randomized clinical control trial. Breast J 2013; 19:114–115. 23241012 10.1111/tbj.12060

[9] Modaghegh MHS, Soltani E. A newly designed SIPC device for management of lymphoedema. Indian J Surg 2010; 72:36–40. 10.1007/s12262-010-0006-7 PMC3452538 23133201

[10] Lee N, Wigg J, Pugh S, Barclay J, Moore H. Lymphoedema management with the LymphFlow advance pneumatic compression pump. Br J Community Nurs 2016; 21:13–19. 10.12968/bjcn.2016.21.Sup10.S13 27715144

[11] Muluk SC, Hirsch AT, Taffe EC. Pneumatic compression device treatment of lower extremity lymphedema elicits improved limb volume and patient-reported outcomes. Eur J Vasc Endovasc Surg 2012; 46:480–487. 10.1016/j.ejvs.2013.07.012 23973278

[12] Dunn N, Williams EM, Fishbourne M, Dolan G, Davies JH. Home management of lower limb lymphoedema with an intermittent pneumatic compression device: A feasibility study. Pilot Feasibility Stud 2019; 5:113. 31583112 10.1186/s40814-019-0496-4 PMC6767651

[13] Lymphoedema Framework. Best practice management of lymphoedema. 2006. Available at: https://tinyurl.com/y8hhd66c (accessed September 27, 2021).

[14] Efird J. Block randomisation with randomly selected block sizes. Int J Environ Res Public Health 2011; 8:15–20. 21318011 10.3390/ijerph8010015 PMC3037057

[15] Williams AF, Whitaker J. Measuring change in limb volume to evaluate lymphoedema treatment outcome. EWMA J 2015; 15:27–32.

[16] Devoogdt N, De Groef A, Hendrickx A, et al. Lymphoedema Functioning, Disability and Health Questionnaire for Lower Limb Lymphoedema (Lymph-ICF-LL): Reliability and validity. Phys Ther 2014; 94:705–721. 24415775 10.2522/ptj.20130285

[17] Zaleska M, Olszewski WL, Durlik M. The effectiveness of intermittent pneumatic compression in long-term therapy of lymphedema of lower limbs. Lymphat Res Biol 2014; 12:103–109. 24927065 10.1089/lrb.2013.0033 PMC4062105

[18] Tran K, Argaez, C. Intermittent Pneumatic Compression Devices for the Management of Lymphedema: A Review of Clinical Effectiveness and Guidelines. Ottawa (ON): Canadian Agency for Drugs and Technologies in Health; 2012. 29553689

[19] Collins C, Mortimer P, D'Ettorre H, A'Hern R, Moskovic E. Computed tomography in the assessment of response to limb compression in unilateral lymphoedema. Clin Radiol 1995; 50:541–544. 7656520 10.1016/s0009-9260(05)83188-5

[20] Greene AK, Meskell P. The impact of lower limb chronic oedema on patients' quality of life. Int Wound J 2017; 14:561–568. 27489034 10.1111/iwj.12648 PMC7949854

[21] Fu MR, Ridner SH, Hu SH. Psychosocial impact of lymphoedema: A systematic review of the literature from 2004 to 2011. Psychooncology 2013; 32:1466–1468. 10.1002/pon.3201 PMC4153404 23044512

[22] Blumberg SN, Berland T, Rockman C, Mussa F, Brooks A, Cayne N, Maldonado T. Pneumatic compression improves quality of life in patients with lower-extremity lymphedema. Ann Vasc Surg 2016; 30:40–44. 26256706 10.1016/j.avsg.2015.07.004

[23] Patsopoulos NA. A pragmatic view on pragmatic trials. Dialogues Clin Neurosci 2011; 13:217–224. 21842619 10.31887/DCNS.2011.13.2/npatsopoulos PMC3181997

